# External validation of ^18^F-FDG PET-based radiomic models on identification of residual oesophageal cancer after neoadjuvant chemoradiotherapy

**DOI:** 10.1097/MNM.0000000000001707

**Published:** 2023-05-03

**Authors:** Maria J. Valkema, Roelof J. Beukinga, Avishek Chatterjee, Henry C. Woodruff, David van Klaveren, Walter Noordzij, Roelf Valkema, Roel J. Bennink, Mark J. Roef, Wendy Schreurs, Michail Doukas, Sjoerd M. Lagarde, Bas P.L. Wijnhoven, Philippe Lambin, John T.M. Plukker, J. Jan B. van Lanschot

**Affiliations:** a Department of Surgery, Erasmus MC Cancer Institute, Rotterdam; b Medical Imaging Centre, Department of Nuclear Medicine and Molecular Imaging, University of Groningen, University Medical Centre Groningen, Groningen; c Department of Precision Medicine, GROW- School for Oncology, Maastricht University; d Department of Radiology and Nuclear Imaging, GROW - School for Oncology, Maastricht University Medical Centre, Maastricht; e Department of Public Health, Erasmus University Medical Centre; f Department of Radiology and Nuclear Medicine, Erasmus MC Cancer Institute, Rotterdam; g Department of Radiology and Nuclear Medicine, Amsterdam University Medical Centre, Amsterdam; h Department of Nuclear Medicine, Catharina Hospital Eindhoven, Eindhoven; i Department of Nuclear Medicine, Zuyderland Medical Centre, Heerlen; j Department of Pathology, Erasmus MC Cancer Institute, Rotterdam; k Department of Surgical Oncology, University Medical Centre Groningen, Groningen, The Netherlands

**Keywords:** ^18^F-FDG PET/CT, active surveillance, neoadjuvant chemoradiotherapy, oesophageal cancer, radiomics

## Abstract

Detection of residual oesophageal cancer after neoadjuvant chemoradiotherapy (nCRT) is important to guide treatment decisions regarding standard oesophagectomy or active surveillance. The aim was to validate previously developed ^18^F-FDG PET-based radiomic models to detect residual local tumour and to repeat model development (i.e. ‘model extension’) in case of poor generalisability.

**Methods:**

This was a retrospective cohort study in patients collected from a prospective multicentre study in four Dutch institutes. Patients underwent nCRT followed by oesophagectomy between 2013 and 2019. Outcome was tumour regression grade (TRG) 1 (0% tumour) versus TRG 2-3-4 (≥1% tumour). Scans were acquired according to standardised protocols. Discrimination and calibration were assessed for the published models with optimism-corrected AUCs >0.77. For model extension, the development and external validation cohorts were combined.

**Results:**

Baseline characteristics of the 189 patients included [median age 66 years (interquartile range 60–71), 158/189 male (84%), 40/189 TRG 1 (21%) and 149/189 (79%) TRG 2-3-4] were comparable to the development cohort. The model including cT stage plus the feature ‘sum entropy’ had best discriminative performance in external validation (AUC 0.64, 95% confidence interval 0.55–0.73), with a calibration slope and intercept of 0.16 and 0.48 respectively. An extended bootstrapped LASSO model yielded an AUC of 0.65 for TRG 2-3-4 detection.

**Conclusion:**

The high predictive performance of the published radiomic models could not be replicated. The extended model had moderate discriminative ability. The investigated radiomic models appeared inaccurate to detect local residual oesophageal tumour and cannot be used as an adjunct tool for clinical decision-making in patients.

## Introduction

Standard treatment for patients with locally advanced oesophageal cancer is neoadjuvant chemoradiotherapy (nCRT) followed by oesophagectomy 6–14 weeks after nCRT [1]. Approximately 30% of patients have a pathologically complete response (pCR) in the resection specimen [2]. Based on this finding, not all patients might require oesophagectomy after nCRT. The possibility of active surveillance for patients with a clinically complete response after nCRT is currently investigated [3,4]. During active surveillance, surgery is offered only when locoregional tumour is detected during clinical response evaluations without evidence of distant metastases on ^18^F-FDG PET/CT. The combination of endoscopy with bite-on-bite biopsies and endoscopic ultrasound with fine-needle aspiration (EUS-FNA) of suspected lymph nodes is 90% sensitive to detect >10% locoregional residual tumour [5]. Further optimisation of tumour detection after nCRT might contribute to improved selection of patients for active surveillance.

High-throughput quantitative imaging, known as radiomics, has been proposed for diagnosis, response evaluation, and prognostication in various types of cancer [6]. Previously, for oesophageal cancer patients who underwent nCRT, internally validated diagnostic prediction models have been developed using pre- and post-treatment ^18^F-FDG PET radiomic features and clinical variables to identify patients with pCR at the primary tumour site [7]. The six best performing models all included one post-treatment radiomic feature plus clinical tumour (cT) stage. An optimism-corrected area under the receiver operating characteristic curve (AUC) of 0.81 was achieved. Such prediction models could potentially be used as a non-invasive add-on tool to the current diagnostic set for clinical response evaluations after nCRT.

The previously developed models have not been externally validated, meaning that they have not been evaluated in patients treated in different hospitals. It is thus unknown whether the models are useful in the clinical setting. The aim of the present study was to externally validate the previously developed models and to explore the possibility of model redevelopment in case of poor generalisability.

## Patients and methods

### Study design

This is a retrospective (TRIPOD type 4) [8] external validation study. Medical ethical approval was obtained for conduct of this study (MEC-2019-0227). All patients provided written informed consent. The Standards for Reporting Diagnostic Accuracy checklist [9], Radiomics reporting guidelines of the Image Biomarker Standardisation Initiative (IBSI) [10] and the radiomic quality score [6] for this study are provided in Supplemental Tables 1-3, Supplemental digital content 1, http://links.lww.com/NMC/A248.

### Patients

The validation cohort included patients who were identified from the databases of the pre–Surgery As Needed for Oesophageal cancer (preSANO) trial and the surgery arm of the SANO trial [3,5]. All patients were referred to standard oesophagectomy 6–14 weeks after CROSS [2] chemoradiotherapy between 2013 and 2019 in four high-volume Dutch institutes. Patients had ^18^F-FDG avid tumours and had a pre-treatment radiotherapy planning CT scan and a post-treatment ^18^F-FDG PET/low dose CT scan 6–12 weeks after nCRT available. The timing of the ^18^F-FDG PET/CT scan within the preSANO trial and SANO trial was dependent on whether residual tumour was detected in the oesophagus after nCRT. As part of these study protocols, patients underwent clinical response evaluations after nCRT. The first clinical response evaluation was performed at 4–6 weeks after nCRT using endoscopy with biopsies. When residual tumour was detected in the oesophagus, an ^18^F-FDG PET/CT scan was performed to exclude distant metastases prior to oesophagectomy. When no residual tumour was detected, a second clinical response evaluation was scheduled after 4–6 weeks. This clinical response evaluation comprised ^18^F-FDG PET/CT, followed by endoscopy with biopsies and EUS-FNA of suspected lymph nodes. In absence of distant disease, patients were referred to standard oesophagectomy.

### Imaging protocols

^18^F-FDG PET/CT scans were acquired according to EARL-1 guidelines [11], similarly to the development cohort [7]. Details on scanner types and imaging protocols are listed in Supplemental Table 2, Supplemental digital content 1, http://links.lww.com/NMC/A248.

### Assessment of clinical and outcome parameters

Clinical staging was performed using the 7^th^ edition of the tumour, node, metastasis (TNM) staging system [12]. Outcome was defined as the response to nCRT at the primary tumour bed according to the Chirieac tumour regression grade (TRG) system [13]: TRG 1, 0% tumour; TRG 2, 1–10% tumour; TRG 3, 11–50% tumour; TRG 4, >50% tumour. TRG scores were assessed by dedicated upper GI pathologists (guided by M.D., with >10 years of expertise) and were dichotomised as TRG 1 versus TRG 2-3-4.

### Radiomic workflow

The radiomic workflow was performed according to the methodology as applied earlier [7] (Supplemental Table 2, Supplemental digital content 1, http://links.lww.com/NMC/A248). In brief, primary tumour delineations, excluding lymph nodes, were based on gross tumour volumes available from radiotherapy CT scans. These volumes were transposed onto the low dose CT and ^18^F-FDG PET scans using the resultant registration vectors. This determined the cranial and caudal borders of the primary tumour area on the post-treatment ^18^F-FDG PET/CT scans. Following, the tumour delineations at the left and right sides of the oesophagus were adapted manually to match the contour of the oesophagus, to adjust for tumour regression after nCRT using MIM Software version 7.1.3 (MIM Software Inc., Cleveland, OH, USA) in consensus by two investigators (R.J.B., who performed tumour delineations in the development cohort, and M.J.V). A threshold method was deliberately omitted since this was inaccurate in patients with a major metabolic response after nCRT. ^18^F-FDG PET scans were converted to SUV and corrected for serum glucose. Voxels were resampled to dimensions of 2 × 2 × 2 mm to obtain the same uniform isotropic voxel grid as in the development cohort. The same feature set as used before, comprising 101 radiomic features (Supplemental Table 4, Supplemental digital content 1, http://links.lww.com/NMC/A248), was calculated using an in-house developed software in Matlab 2014b (Mathworks, Natick, MA, USA) with definitions according to the IBSI guidelines.

### External validation

External validation was performed for the six internally validated models with optimism-corrected AUCs >0.77. For every patient in the external validation cohort, the logarithm of the odds ratio was calculated using the previously reported regression coefficients [7] as follows:


$$LogO=intercept+coefficient_{cT1-2\;or\;cT3-4a}\;+coefficient_{radiomic\;feature}*radiomic\;feature\;value$$

The probability of a patient having TRG 1 was calculated with the formula:


$$probability=\frac{\exp\left(LogO\right)}{1+\text{exp}\left(LogO\right)}$$

### Model extension

The previously developed models were revised to detect TRG 2-3-4, *that is*, ‘model extension’ [14], based on the development cohort and the external validation cohort combined. This enabled re-estimating (new combinations of) predictors in a larger sample size. Only post-treatment ^18^F-FDG PET features were considered based on the previous study [7].

**Fig. 1:**
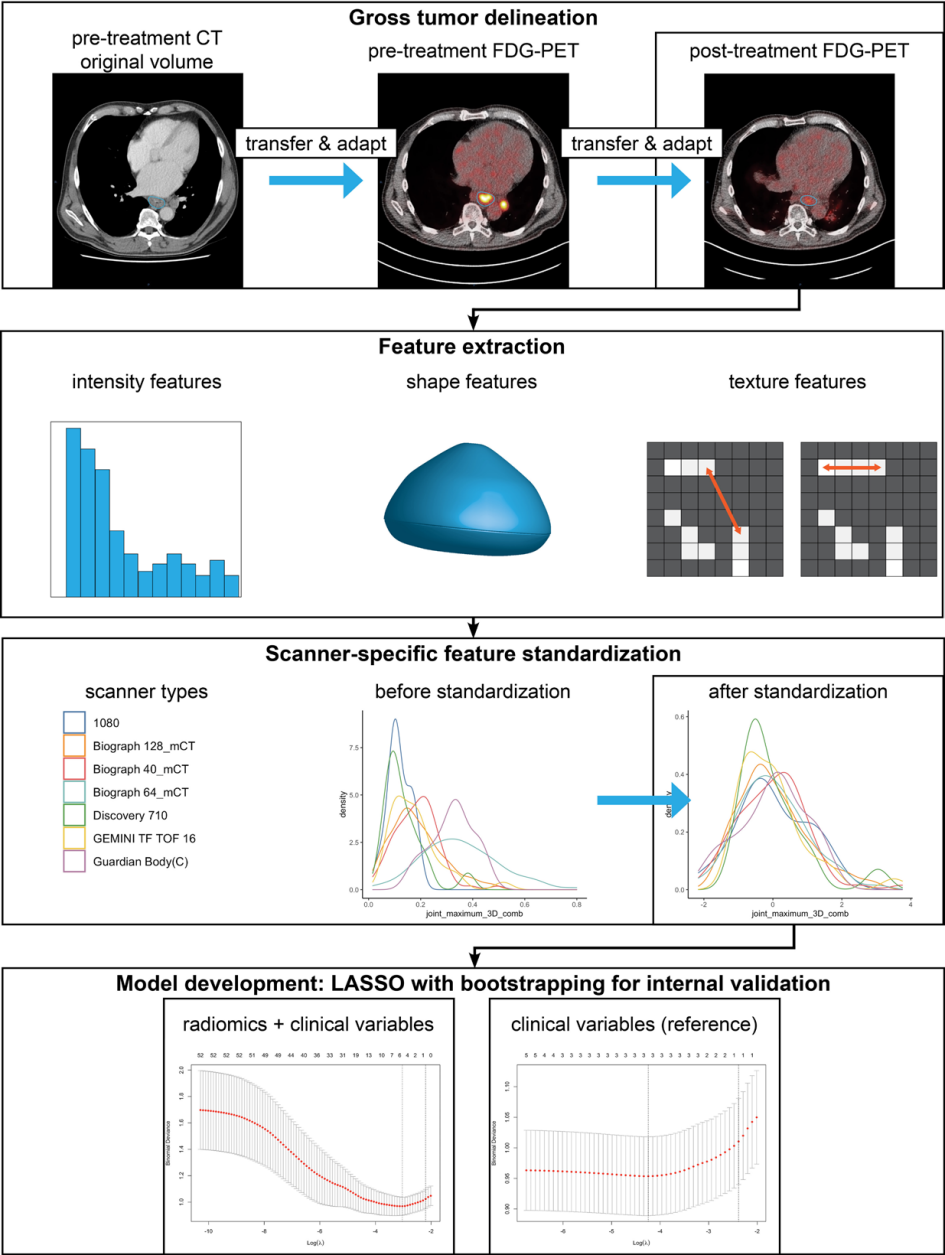
Radiomic workflow for model extension. In brief, radiomic features were calculated from gross volumes as delineated on post-treatment ^18^F-FDG PET/CT scans. Radiomic features were standardised per scanner model. After removal of highly correlated radiomic features, LASSO models were developed with bootstrapping for internal validation. A LASSO model based on radiomics plus clinical variables was compared to a reference LASSO model based on clinical variables only.

The workflow for model extension is shown in Fig. 1. Features were standardised per scanner model, with features set at mean 0 and SD 1 (Supplemental Appendix 1, Supplemental digital content 1, http://links.lww.com/NMC/A248) [15]. The radiomic feature set was complemented with the variables cT stage, clinical lymph node (cN) stage, age, sex and histology [16]. Highly correlated variables were removed at a pair-wise absolute correlation cut-off of 0.9. With the remaining variables, a least absolute shrinkage and selection operator (LASSO) model was developed with bootstrapping (200 bootstrap samples) [17]. A bootstrapped LASSO model limited to only clinical variables was developed for reference. For comparison, another radiomic workflow was applied [18]. Using stratified random subsampling (details in Supplemental Appendix 1, Supplemental digital content 1, http://links.lww.com/NMC/A248), performance estimates of three simple linear models and a random forest classifier were explored over 100 training and validation partitions of the dataset.

In case of insufficient model performance, model extension was repeated to distinguish substantial residual tumour (TRG 3-4) from no or minor residual tumour (TRG 1-2). This endpoint was chosen since it is in line with current research on active surveillance and might be appropriate since small residual tumour (1–10%) might not be detectable on ^18^F-FDG PET/CT [3,5].

### Statistical analysis

Continuous variables were presented as median with interquartile range (IQR), and comparisons were performed with a parametric Student’s *t*-test or a nonparametric Mann–Whitney *U* test. Categorical variables were reported with frequencies and percentages and were compared using a parametric chi-squared test or nonparametric Fisher’s exact test. Two-sided *P* values <0.05 were considered statistically significant.

Discrimination of models was measured with AUC (ideal value: 1). Calibration was assessed for externally validated models using the calibration slope (ideal value: 1) and intercept (ideal value: 0). For every model, at a probability threshold chosen to obtain 90% sensitivity [5], corresponding specificity, positive predictive value, negative predictive value and accuracy were reported.

Statistical analysis was performed using R version 4.0.4 (www.r-project.org). Code for data analysis was made publicly available via github.com/mjvalkema/esophageal-cancer-radiomics.

## Results

### Patients

**Fig. 2:**
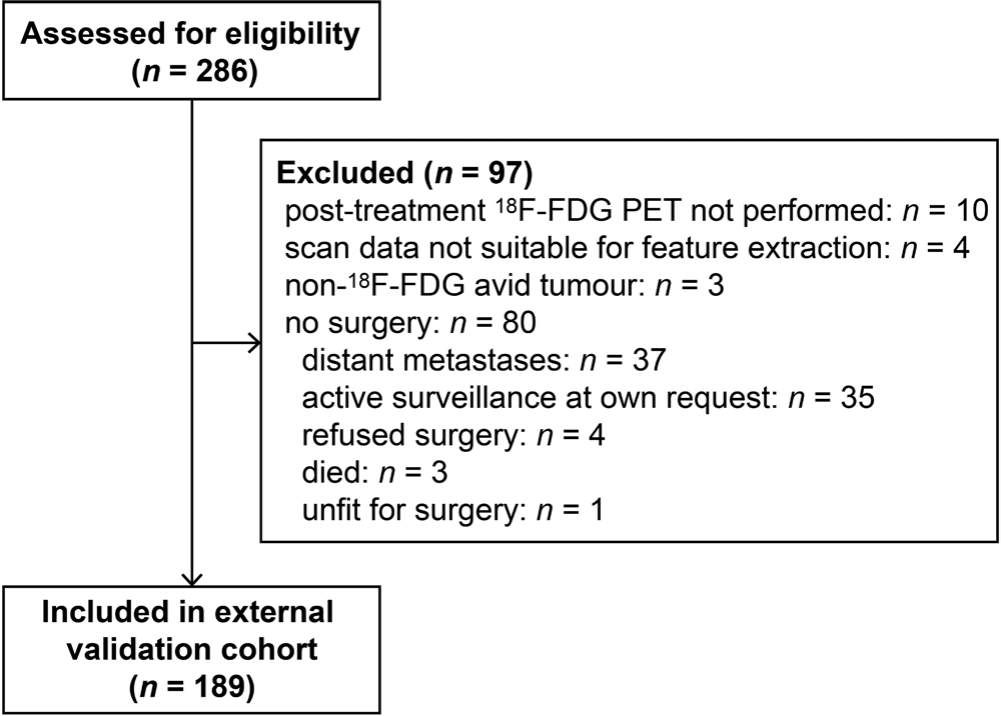
Flow diagram of study patients of the external validation cohort.

Some 189 were included in the external validation cohort (Fig. 2). Patients received treatment between July 2013 and May 2019 in the Erasmus University Medical Centre, Rotterdam (116 of 189 patients, 61%), the Amsterdam University Medical Centre, Amsterdam (28 of 189 patients, 15%), the Catharina Hospital Eindhoven, Eindhoven (25 of 189 patients, 13%) or the Zuyderland Medical Centre, Heerlen (20 of 189 patients, 11%). ^18^F-FDG PET/CT scans were acquired at a median of 10.3 weeks after nCRT (IQR 8.1–11.2). Demographics and tumour characteristics were comparable between the validation cohort and the development cohort, except for cN0/cN+ stage (cN0 stage in 67 of 189 patients (35%) versus 15 of 73 patients (21%) respectively, *P* = 0.024) (Table 1). Two patients had unresectable tumour (T4b), and were assigned an arbitrary ‘TRG 4’ score. The outcome TRG 1 versus TRG 2-3-4 was equally distributed between the development and external validation cohorts (TRG 1 in 40 of 189 patients (21%) versus 16 of 73 patients (22%), *P =* 1.00).

**Table 1:** Overview of study, patient and tumour characteristics

	Development cohort (*n* = 73)	External validation cohort (*n* = 189)	*P*-value^a^
Data collection period	2014–2017	2013–2019	-
Study design	Consecutive patients from 1 prospective cohort	Consecutive patients from 2 prospective cohorts	-
Setting	1 high-volume Dutch hospital	4 high-volume Dutch hospitals	-
Treatment	nCRT according to CROSS + surgery	nCRT according to CROSS + surgery	-
Timing of scan after nCRT	6–12 weeks after nCRT	6–8 weeks after nCRT	-
Outcome	TRG according to Mandard [19]	TRG according to Chirieac [13]	-
Sex			0.54
Male	58 (79)	158 (84)	-
Female	15 (21)	31 (16)	-
Age	63 [59–69]	66 [60–71]	0.35
Histology			0.057
Adenocarcinoma	65 (89)	147 (78)	
Squamous cell carcinoma	8 (11)	42 (22)	
Clinical T-stage			0.10^b^
1	0 (0)	1 (0.5)	
2	9 (12)	41 (22)	
3	59 (81)	140 (74)	
4a	5 (7)	7 (4)	
Clinical N-stage			**0.024** c
0	15 (21)	67 (35)	
1	32 (44)	79 (42)	
2	22 (30)	39 (21)	
3	4 (6)	3 (2)	
Nx	0 (0)	1 (0.5)	
TRG			1.00^d^
1	16 (22)	40 (21)	
2	19 (26)	54 (29)	
3	23 (32)	52 (28)	
4-5	15 (20)	43 (23)	

Continuous values are median [interquartile range] and categorical values are *n* (%) unless denoted otherwise. Numbers may not add up to 100% due to rounding.

Bold text denotes *P* < 0.05.

nCRT, neoadjuvant chemoradiotherapy; TRG, tumour regression grade.

a Mann–Whitney *U* test (continuous variables) or chi-squared test (categorical variables).

b Chi-squared test cT1-2 versus cT3-4a.

c Chi-squared test cN0/cNx versus cN+.

d Chi-squared test TRG 1 versus TRG 2-3-4.

### External validation

The six evaluated radiomic features differed between the cohorts with statistical significance, as shown in Supplemental Table 5 and Figure 1, Supplemental digital content 1, http://links.lww.com/NMC/A248.

**Fig. 3:**
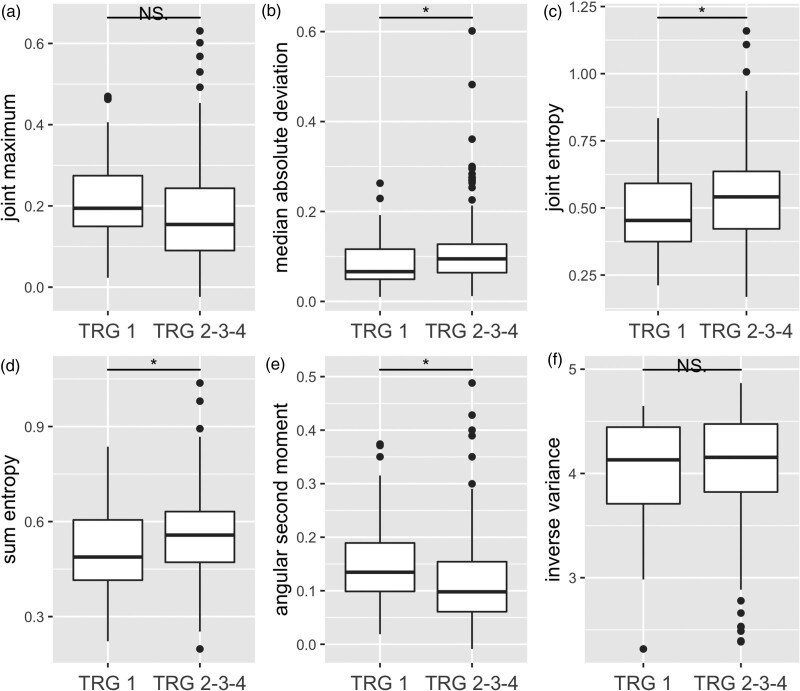
Boxplots for visual comparison of the six radiomic features versus the outcome TRG in the external validation cohort. NS, non-significant; TRG, tumour regression grade. *P < 0.05.

The six prediction models were applied on the external validation dataset. The radiomic feature values of each of the six models versus the outcome of interest are shown in Fig. 3. The extreme effect between outcome and cT stage as seen in the development cohort was less strong in the external validation cohort. Of the nine patients with cT1-2 stage in the development cohort, seven (78%) had complete response (TRG 1). The validation cohort comprised 41 patients with cT1-2 stage of whom 12 (29%) had TRG 1 (*P* = 0.02) (Supplemental Table 6, Supplemental digital content 1, http://links.lww.com/NMC/A248).

**Fig. 4:**
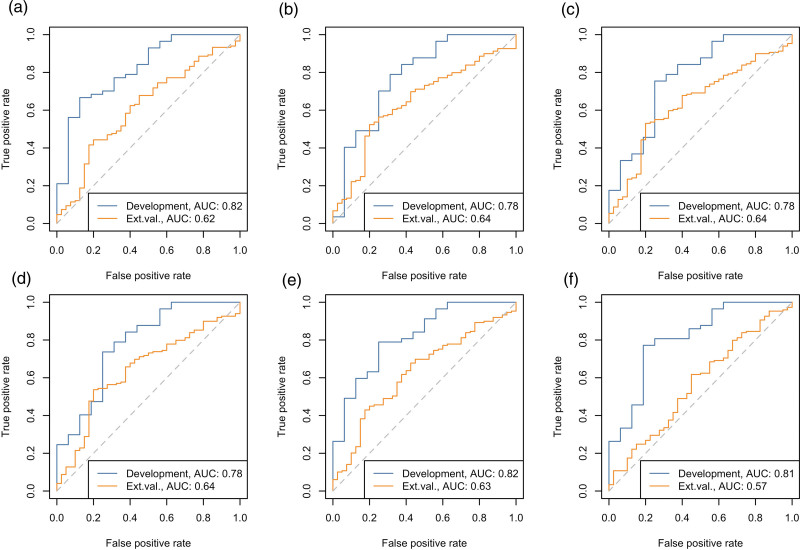
Receiver operating characteristic (ROC) curves for the six externally validated prediction models (a–f) with AUCs >0.77 in the development cohort. The blue line corresponds with the development cohort (*n* = 73) and the orange line with the external validation cohort (*n* = 189). AUC, area under ROC curve; Development, development cohort; Ext. val., external validation cohort.

The performance metrics of the six models in the external validation cohort are shown in Table 2 and Fig. 4. Discriminative performance improved when scans of one vendor were used, and decreased when a subgroup of only adenocarcinoma patients was used (Supplemental Figure 2, Supplemental digital content 1, http://links.lww.com/NMC/A248). The model including cT stage and the feature ‘sum entropy’ had highest AUC [0.64; 95% confidence interval (CI) 0.55–0.73], with a calibration slope of 0.16 (95% CI −0.05 to 0.37) and intercept of 0.48 (95% CI 0.0–0.96) (Table 2). For each of the externally validated models, histograms of the predicted probabilities for TRG outcome are shown in Supplemental Figure 3, Supplemental digital content 1, http://links.lww.com/NMC/A248.

**Table 2:** Performance of the six original models on the external validation cohort (*n* = 189)

Model	Variables in model	AUC (95% CI^a^)	Prob. thresh. b (%)	Sens. (%)	Spec. (%)	PPV (%)	NPV (%)	Acc. (%)	Calibration slope (95% CI^a^)	Calibration intercept (95% CI^a^)
A	cT + joint maximum	0.62 (0.51–0.71)	0.53	90	15	80	29	74	0.15 (−0.06 to 0.35)	−1.18 (−1.66 to −0.69)
B	cT + median absolute deviation	0.64 (0.55–0.73)	0.58	90	12	79	25	74	0.15 (−0.06 to 0.35)	−1.39 (−1.87 to −0.90)
C	cT + joint entropy	0.64 (0.54–0.73)	0.64	90	12	79	25	74	0.16 (−0.05 to 0.36)	−1.66 (−2.14 to −1.18)
D	cT + sum entropy	0.64 (0.55–0.73)	0.18	90	12	79	25	74	0.16 (−0.05 to 0.37)	0.48 (0.0 to 0.96)
E	cT + angular second moment	0.63 (0.53–0.72)	0.12	90	12	79	25	74	0.15 (−0.06 to 0.36)	0.97 (0.49 to 1.44)
F	cT + inverse variance	0.57 (0.46–0.66)	0.75	90	17	80	32	75	0.14 (−0.06 to 0.34)	−2.08 (−2.56 to −1.59)

acc., accuracy; AUC, area under the receiver operating characteristic curve; CI, confidence interval; NPV, negative predictive value; PPV, positive predictive value; prob. thresh., probability threshold; sens., sensitivity; spec., specificity.

a 95% CI was determined with 2000 bootstrap replicates.

b Probability threshold chosen to obtain 90% sensitivity.

### Model extension

Radiomic features were corrected for variations across different scanner models (Supplemental Figures 4–6, Supplemental digital content 1, http://links.lww.com/NMC/A248). Following, 49 variables with pair-wise absolute correlations >0.9 were removed. A LASSO model was developed that included five variables with non-zero coefficients, three of which were radiomic features and the clinical variables cT stage and histology (Supplemental Table 7 and Figure 7, Supplemental digital content 1, http://links.lww.com/NMC/A248). The extended LASSO model yielded an optimism-corrected AUC of 0.65 (uncorrected 0.75) (Table 3). The LASSO model with clinical variables (Supplemental Table 7 and Figure 7, Supplemental digital content 1, http://links.lww.com/NMC/A248) yielded and optimism-corrected AUC of 0.71 (uncorrected 0.73) (Table 3).

**Table 3:** Performance metrics of the extended LASSO model using the combined datasets (*n* = 262)

LASSO model	Endpoint	Internally validated AUC^a^ (apparent AUC)	Prob. thresh. b (%)	Sens. (%)	Spec. (%)	PPV (%)	NPV (%)	Acc. (%)
Radiomic features + clinical variables	TRG 2-3-4	0.65 (0.75)	0.70	90	44	85	55	80
Clinical variables	TRG 2-3-4	0.71 (0.73)	0.64	90	40	85	52	79
Radiomic features + clinical variables	TRG 3-4	0.59 (0.69)	0.44	90	36	58	78	63
Clinical variables	TRG 3-4	0.58 (0.61)	0.40	90	23	54	70	57

acc., accuracy; AUC, area under the receiver operating characteristic curve; LASSO, least absolute shrinkage and selection operator; NPV, negative predictive value; PPV, positive predictive value; prob. thresh., probability threshold; sens., sensitivity; spec., specificity.

a AUC was internally validated using bootstrapping (200 bootstrap samples).

b Probability threshold chosen to obtain 90% sensitivity.

The LASSO modelling strategy was compared to another workflow for radiomic feature selection and model development. The first step of this workflow indicated that a generic radiomic analysis was suitable, as shown in Supplemental Appendix 2 and Figure 8, Supplemental digital content 1, http://links.lww.com/NMC/A248. Performance distributions of the models combining the selected radiomic features (Supplemental Table 8, Supplemental digital content 1, http://links.lww.com/NMC/A248) with clinical variables are shown in Supplemental Table 9, Supplemental digital content 1, http://links.lww.com/NMC/A248. Over the 100 validation datasets, a Naive Bayes classifier achieved a mean AUC of 0.73 (95% CI 0.63–0.84). Since an independent dataset was not available, optimism of this model could not be further investigated.

Models were also trained to detect substantial residual tumour (i.e. TRG 3-4), but this did not result in improved diagnostic accuracy compared to the models trained for the primary endpoint (i.e. TRG 2-3-4) (Table 3 and Supplemental Table 9, Supplemental digital content 1, http://links.lww.com/NMC/A248).

## Discussion

Generalisability of previously developed ^18^F-FDG PET-based radiomic models [7] was not confirmed in this independent validation cohort. The discriminative ability of an extended LASSO model did not outperform the clinical reference model. The evaluated radiomic models in this study appeared insufficient as a potential adjunct tool for clinical decision-making in individual patients.

Several similar studies in oesophageal cancer have been performed, but most of these are limited by small sample sizes [20–23]. The results of the current study are best compared to the study of Van Rossum *et al*. [20], in which radiomic models did not meet a relevant threshold to impact clinical decision-making. Their best performing model, incorporating clinical variables and ^18^F-FDG PET parameters from 217 patients, achieved a corrected *C*-index of 0.77 for prediction of pCR [20].

The performance of the investigated models in this study was evaluated at a probability threshold to obtain 90% sensitivity, which is the benchmark for detection of residual TRG 3-4 tumour using gastroscopy with bite-on-bite biopsies and EUS-FNA of suspected lymph nodes [5]. The resulting specificity of the evaluated models for TRG 2-3-4 detection at this threshold was low. Subsequently, models were trained to detect >10% residual tumour (TRG 3-4) versus 0–10% residual tumour (TRG 1-2). This endpoint may be justified since it might be safe to miss minor residual tumour initially, which then becomes timely detectable at one of the subsequent clinical response evaluations [5]. Furthermore, it might be easier to train a model to select features belonging to substantial residual tumour, which is better visible on ^18^F-FDG PET/CT. However, models trained for the endpoint TRG 3-4 performed marginally better than a model with only clinical variables. Thus, for both endpoints, radiomic features appeared not of added value. This might be interpreted as the inability of the investigated features to capture pathophysiological information. Apparently, the radiomic features fail to encode information about differences in tissue between patients with and without (major) residual tumour after nCRT. This was different than in the derivation sample alone, in which radiomic features, representing, for example, orderliness of the voxels, appeared to distinguish residual tumour from normal tissue (with treatment effects) [7].

The finding that radiomics did not improve detection of residual tumours may not be surprising in the context of a previous study [24]. Qualitative assessment of ^18^F-FDG PET/CT has been shown inaccurate for distinction between residual tumour and inflammation at the primary tumour site at 12 weeks after nCRT. Radiomics was expected to capture more complex information within the image, for example, reflecting underlying biology. Such information did not appear to be retrievable from the investigated imaging dataset.

A strength of this study is the homogeneous multicentre external validation cohort collected from two prospective trials [3,5]. This results in similar scanning protocols and application of the same nCRT regimen across the different institutions. Moreover, quality assessment of tumour delineations was performed with the author of the previous article, and radiomic feature calculation method was kept the same as in the development cohort [7]. Furthermore, another radiomic workflow was demonstrated for model extension (Supplemental Appendix 1, Supplemental digital content 1, http://links.lww.com/NMC/A248) [18], since there might not be a single correct modelling approach [25]. This workflow has the advantage that feature pre-selection is more intuitive to understand and is best used when an independent validation set is available.

The limited generalisability of the published models is probably due to overfitting to the derivation sample. Another possible cause for decreased performance in the validation cohort is unintentional dependency of radiomic features on scanning protocols, scanner models (see Supplemental Figure 2, Supplemental digital content 1, http://links.lww.com/NMC/A248) and tumour delineation methods [15]. Heterogeneity in the assessment of cT stage across different hospitals might also have negatively impacted generalisability of the models. The variable cT stage had a relatively large contribution to the predictions of the six published models (regression coefficient between −2.7 and −2.9) [7].

There are several other limitations of this study. The external validation cohort included patients who underwent ^18^F-FDG PET/CT between 6 and 12 weeks after nCRT, whereas for the development cohort the timing was between 6 and 8 weeks after nCRT. Radiomic feature values might therefore have been less alike between the cohorts because of differences in post--radiotherapy inflammatory effects in these time periods. Unfortunately it was not informative to validate the models on a subgroup of patients who underwent a ^18^F-FDG PET/CT scan until 8 weeks after nCRT. Because of the timing of the ^18^F-FDG PET/CT within clinical response evaluations of the pre-SANO trial [5] and SANO trial [3], this would result in a cohort comprising solely patients with residual tumour. As such, model performance cannot be compared between such a subgroup and the full external validation cohort. With regard to model updating, a limitation is that other classes of features, such as wavelet features, were not considered because these were not available from the previous study [7]. Moreover, non-linear associations may exist between predictors and the outcome of interest. We explored a random forest model for this purpose, but this model easily overfitted (data not shown). A larger sample size would enable further investigation of non-linear relations. Furthermore, despite the use of data from prospective trials, it is plausible that there are variations in application of nCRT, scanning, surgery, and outcome assessment that we could not correct for.

Even though the results of the present study do not improve decision-making after nCRT, they underline the importance of external validation. External validation is warranted to assess whether a developed prediction model applies outside the development setting. The current findings may help to inform the design of future studies to make response evaluations after nCRT more accurate and less invasive [26]. It might be worthwhile to explore a multi-omics approach involving imaging modalities more suitable for tumour recognition (e.g. endoscopy and diagnostic CT) in combination with other biomarkers.

In conclusion, this external validation study in a multicentre external validation cohort could not replicate the high predictive performance of radiomic models incorporating post-treatment ^18^F-FDG PET features and cT stage. Model extension based on the combined cohorts was not successful either. The application of radiomics to ^18^F-FDG PET/CT scans up to 12 weeks after nCRT is of no help in decision-making in individual patients regarding the choice for active surveillance after nCRT. The results underline the necessity to use homogenous imaging datasets during model development and to externally validate a predictive model before it can be applied in broad clinical use.

## Acknowledgements

We are grateful to B.J. Noordman, B.J. van der Wilk and B.M. Eyck for the coordination of study participants from the preSANO and SANO trials, S. Sanduleanu for helping organising the imaging dataset, L.R. de Ruiter for writing code for radiomic analyses, and W. Schillemans, J. Nuyttens and J. Buijsen for providing the radiotherapy imaging data.

### Conflicts of interest

M.J.V. and J.J.B.v.L. disclose scientific grants from the KWF Dutch Cancer Society (funding of the SANO trial, project number 10825, and the preSANO trial, project number EMCR-2014-7430) and The Netherlands Organisation for Health Research and Development (ZonMw) (funding of the SANO trial, project number 843004104). P.L. discloses, within and outside the submitted work, grants/sponsored research agreements from Radiomics SA, ptTheragnostic/DNAmito and Health Innovation Ventures. He received an advisor/presenter fee and/or reimbursement of travel costs/consultancy fee and/or in-kind manpower contribution from Radiomics SA, BHV, Merck, Varian, Elekta, ptTheragnostic, BMS and Convert Pharmaceuticals. P.L. has minority shares in the companies Radiomics SA, Convert Pharmaceuticals, Comunicare and LivingMed Biotech, and he is co-inventor of two issued patents with royalties on radiomics (PCT/NL2014/050248, PCT/NL2014/050728), licensed to Radiomics SA, and one issued patent on mtDNA (PCT/EP2014/059089), licensed to ptTheragnostic/DNAmito, three non-patented inventions (softwares) licensed to ptTheragnostic/DNAmito, Radiomics SA and Health Innovation Ventures and three non-issued, non-licensed patents on Deep Learning-Radiomics and LSRT (N2024482, N2024889, N2024889). He confirms that none of the above entities or funding sources were involved in the preparation of this article. H.C.W. discloses minority shares in the company Radiomics SA, unrelated to this work. For the remaining authors, there are no conflicts of interest.

## Supplementary Material


