# Supplementary material for: External validation of 18F-FDG PET-based radiomic models on identification of residual oesophageal cancer after neoadjuvant chemoradiotherapy
Source: Nucl Med Commun. 2023 May 3;44(8):709–18. doi: 10.1097/MNM.0000000000001707 (PMC10337315; doi:10.1097/MNM.0000000000001707)
Supplement: Supplementary file 1 [file nmc-44-709-s001.pdf]

DIGITAL SUPPLEMENTAL INFORMATION

Table of Contents

Appendix 1. Methods model extension 2

Availability of code 2

Data preparation for model extension 2

Scanner-specific standardization 2

ComBat harmonization 3

Methods model extension 3

Clearance step 4

Feature selection with stratified random subsampling 5

Appendix 2. Results model extension 6

Clearance step 6

Feature selection step and model development 6

Supplemental Tables 7

Supplemental Figures 24

Appendix 1. Methods model extension

Availability of code

The code, including all R packages used for this study, is available via: github.com/mjvalkema/esophageal-cancer-radiomics.

Data preparation for model extension

The previously developed models were revised based on the combined datasets. This allowed consideration of additional and other combinations of predictors in a larger sample size, *i.e.* “model extension” [1]. By combining the datasets, model generalizability to unseen data, *e.g.* from other institutes, is expected to be improved, which is important for eventual transferability of a radiomic prediction model to clinical practice. The development of radiomic models based on multicentre data however is expected to result in a somewhat decreased performance. In addition, radiomic feature values are known to be dependent on different scanner manufacturers, scanner types, acquisition protocols, post-reconstruction methods and tumour delineation methods [2, 3]. Normalization methods are often applied to limit fluctuations in feature values. Normalization of features generally contributes to better performance of prediction models [2].

Scanner-specific standardisation

Instead of applying normalization on the entire dataset of multiple institutions, a methodology has been proposed to standardise features (mean 0 and standard deviation 1) separately for each institute in the dataset (*i.e.* institution-specific standardisation) [2]. In contrast to normalization through rescaling, this methodology preserves outlier values. Since in the current dataset multiple scanner types were used per institute (Supplemental Table 2), features were standardised for every scanner model separately (*i.e.* scanner-specific standardisation). Scanner-specific standardisation was done for scanners on which a minimum of eight patients were scanned, based on the numbers of scanner types in the current dataset. It was not considered sensible to include the scanners with fewer patients than eight (in the present dataset there are scanners on which ≤3 patients were

scanned, see Supplemental Table 2).

ComBat harmonization

The effect of scanner-specific standardisation was explored in relation to another frequently used normalization method called ComBat harmonization. ComBat harmonization sets features of different batches in a comparable range while biological information is preserved [4]. The precise workflow for ComBat harmonization has been described in a previous study and was shown to facilitate multicentre radiomic studies using PET imaging [3]. We explored whether ComBat harmonization was able to adjust for centre effects in the present dataset. It was applied using the R package “neuroComBat”, with non-parametric settings. For the “batch” parameter in this function, the scanner model type was used; for the “mod” parameter, the outcome of interest was used. The effect of both normalization methods was visually compared using boxplots for each of the six features as incorporated in the six externally validated prediction models. We did not proceed with ComBat harmonization because some 43 patients had to be excluded to meet the requirement for 20-30 patients per batch.

Methods model extension

For model extension, all features in the combined dataset were normalised using scanner- specific standardisation. This method successfully corrected scanner differences (Supplemental Figure 4, Supplemental Figure 5, Supplemental Figure 6), does not require at least 20-30 patients per batch, and is intuitive to understand. Model extension was performed using least absolute shrinkage and selection operator (LASSO) with bootstrapping (200 bootstrap samples) for internal validation. The function “cv.glmnet” in R (nfolds = 10) was used to determine the value of lambda that minimises binomial deviance.

Further details on model building using LASSO are provided within the manuscript. The LASSO workflow was compared to another modelling strategy for high-dimensional radiomic datasets that has been published previously [5]. The strategy is briefly explained in the following paragraphs ("clearance step" and "feature selection with stratified random subsampling").

Clearance step

The clearance step is not a feature selection step but identifies all features that might have potential prognostic capability. First, ^{18}F-FDG PET features without any meaningful information are removed from the feature set: the minimum relative variation that a feature should have, defined by the standard deviation of the feature divided by the mean value of the feature, was required to be >0.05. Furthermore, the maximum fraction of patients for whom a feature can have the same value was set to 0.3.

Following, the feature dataset was randomly stratified into two equal parts (S_1 and S_2); this was done 100 times. Outcome distributions were kept similar in every split.

For a certain split i, all features that had an absolute linear correlation with the clinical outcome >0.2 in $S_{1,i}$ were considered. For each split, a scatter plot of these features, showing the correlation in $S_{1,i}$ versus the correlation in $S_{2,i}$ was made; the linear correlation coefficient of that plot, R_p, was calculated. Since there were 100 splits, this produced 100 values of R_p. The mean of this distribution was computed. The ideal value of the mean correlation is 1. If the mean R_p is between 0 and 0.5, it suggests that a few features might indeed be predictive, but by casting a wide net, too many useless features are being explored. If the mean R_p is a negative number, it indicates that the features that have a high correlation with outcome in S_1 have a low correlation with outcome in S_2. Thus in this case, there are no reliable feature correlations with outcome, and the imaging modality is best avoided. If the mean R_p is greater than 0.5, then the imaging modality might be a productive one [5]. The relative difference in R between S_1 and S_2 was also calculated, and averaged

over the 100 splits, *i.e.* “mean relative uncertainty”: mean($1 – R(S_{2,i}) / R(S_{1,i})$). The ideal value is close to 0.

Feature selection with stratified random subsampling

The dataset was randomly stratified into training and validation sets (2:1 ratio), repeated 100 times to derive performance distributions [5]. Outcome proportions were kept similar in the training and validation datasets. Only features with median AUCs >0.60 in the 100 training sets were retained. Of the remaining features, pairwise feature elimination was performed: if the absolute correlation between two features was > 0.7, the one with lower AUC was removed. This produced a reduced radiomic feature set to which clinical variables were added [6].

Three simple linear models, a logistic regression, Naïve Bayes (selected radiomic features plus clinical variables) and a class-balanced linear support vector machine (SVM) (selected radiomic features plus clinical variables), were explored, chosen because of low risk of overfitting [2]. To avoid overfitting with the logistic regression model for detection of TRG 2- 3-4, the number of radiomic features was limited to the two features with highest AUCs in univariate analysis. To avoid problems with unbalanced data in the SVM model, outcomes for TRG 1 versus TRG 2-3-4 were balanced with weighting. Performance distributions of the three models were shown over the 100 training and validation datasets. Since an independent validation set was not available, optimism of the three models could not be further investigated.

For comparison, the dataset with radiomic and clinical variables was also entered in a non- linear model. A random forest classifier was trained with balanced data in each bootstrap sample with the function RandomForestClassifier as implemented in Sklearn in Python 3.7.4. The following settings were applied: maximum depth = 3, maximum number of features = “auto”, number of trees = 100, bootstrapping = TRUE, class weight = “balanced subsample”.

A random state was chosen to enable replication of the model. Hence, for the random forest classifier, independent validation could not be performed since an independent dataset was not available.

Appendix 2. Results model extension

Clearance step

No features in the post-nCRT ^{18}F-FDG PET dataset were removed based on the threshold for minimum relative variation or based on having the same value to often. For detection of TRG 2-3-4, the mean R_p was 0.88 ± 0.11 (required to be at least >0.5, ideal value 1). The mean relative uncertainty was 0.47 ± 0.34 (required to be at least < 1, ideal value 0) (Supplemental Figure 8). These metrics were sufficient to proceed with the rest of the machine learning workflow [5]. For detection of TRG 3-4, the mean R_p was 0.74 ± 0.34, the mean relative uncertainty was 0.64 ± 0.35.

Feature selection step and model development

Features with AUCs >0.60 retained after the feature selection step are shown in Supplemental Table 8. These radiomic features were combined with the clinical variables cT, clinical lymph node stage (cN), age, sex and histology [6]. The variables were explored using three linear models and a random forest classifier. The mean performance metrics with 95% confidence intervals over the 100 training and validation datasets (2:1 ratio) are shown in Supplemental Table 9.

Supplemental Tables

Supplemental Table 1

STARD 2015 checklist (available from: equator-network.org/reporting-guidelines/stard)

Section and topic	No.	Item	
**TITLE OR ABSTRACT**			
	1	Identification as a study of diagnostic accuracy using at least one measure of accuracy (such as sensitivity, specificity, predictive values, or AUC)	
ABSTRACT			
	2	Structured summary of study design, methods, results, and conclusions (for specific guidance, see STARD for Abstracts)	☑
INTRODUCTION			
	3	Scientific & clinical background, including the intended use and clinical role of the index test	☑
	4	Study objectives and hypotheses	☑
METHODS			
Study design	5	Whether data collection was planned before the index test and reference standard were performed (prospective study) or after (retrospective study)	☑
Participants	6	Eligibility criteria	☑
	7	On what basis potentially eligible participants were identified (such as symptoms, results from previous tests, inclusion in registry)	☑
	8	Where and when potentially eligible participants were identified (setting, location and dates)	☑
	9	Whether participants formed a consecutive, random or convenience series	☑
Test methods	10a	Index test, in sufficient detail to allow replication	☑
	10b	Reference standard, in sufficient detail to allow replication	☑
	11	Rationale for choosing the reference standard (if alternatives exist)	☑
	12a	Definition of and rationale for test positivity cut-offs or result categories of the index test, distinguishing pre-specified from exploratory	☑
	12b	Definition of and rationale for test positivity cut-offs or result categories of the reference standard, distinguishing pre-specified from exploratory	☑
	13a	Whether clinical information and reference standard results were available to the performers/readers of the index test	☑ in Supplemental Data (Supplemental Table 2)
	13b	Whether clinical information and index test results were available to the assessors of the reference standard	N/A, outcome assessment was done before the radiomic workflow was conducted
Analysis	14	Methods for estimating or comparing measures of diagnostic accuracy	☑
	15	How indeterminate index test or reference standard results were handled	☑
	16	How missing data on the index test and reference standard were handled	N/A, in the validation cohort there were no missing data for radiomic features or outcome
	17	Any analyses of variability in diagnostic accuracy, distinguishing pre-specified from exploratory	☑
	18	Intended sample size and how it was determined	N/A
RESULTS			

Participants	19	Flow of participants, using a diagram. Include the figure number (preferably figure 1) or page number	☑
	20	Baseline demographic and clinical characteristics of participants	☑
	21a	Distribution of severity of disease in those with the target condition	☑
	21b	Distribution of alternative diagnoses in those without the target condition	N/A
	22	Time interval and any clinical interventions between index test and reference standard	☑
Test results	23	Cross tabulation of the index test results (or their distribution) by the results of the reference standard	☑
	24	Estimates of diagnostic accuracy and their precision (such as 95% confidence intervals)	☑
	25	Any adverse events from performing the index test or the reference standard	N/A
DISCUSSION			
	26	Study limitations, including sources of potential bias, statistical uncertainty, and generalizability	☑
	27	Implications for practice, including the intended use and clinical role of the index test	☑
OTHER INFORMATION			
	28	Registration number and name of registry	N/A
	29	Where the full study protocol can be accessed	N/A
	30	Sources of funding and other support; role of funders	☑

Supplemental Table 2. Radiomic feature calculation methodology for the external validation cohort, reported according to the guidelines of the Image Biomarker Standardisation Initiative (IBSI)

Topic	Item	Description of the item in the external validation cohort	Result in external validation cohort (*n* = 189) Reported as *n* (%) or median [IQR]
**Patient**			
**Region of interest**	Region of interest	Gross tumour volume of the primary tumour	
**Patient preparation**	Instructions given to patients prior to image acquisition	At least 6 hours of fasting and 2 litres of pre-hydration, and being in resting conditions before scanning	
	Administration of drugs to the patient prior to image acquisition	-	
	Describe use of specific equipment for patient comfort during scanning	-	
**Radioactive tracer**	Which radioactive tracer	^{18}F-FDG	
	Administration method	Intravenous administration	
	Injected activity at administration	2.3 MBq/kg	201 MBq [178, 253]
	Uptake time prior to image acquisition	60 ± 5 minutes	60 minutes [58, 64]
	How competing substance levels were controlled	Fasting before scanning; blood glucose level was measured before scanning; SUV_{max} was corrected for blood glucose	5.7 mmol/L [5.2, 6.3]
**Contrast agent**	Which contrast agent was administered	-	
**Comorbidities**	Do patients have comorbidities that affect imaging	-	
**Acquisition**			
**Acquisition protocol**	Was a standard imaging protocol used	European Association of Nuclear Medicine guidelines version 1.0 [7]	
**Scanner type**	Vendors	SIEMENS ®	130 (69)
		GE Healthcare Systems ®	20 (11)
		Philips ®	39 (21)
	Scanner types	SIEMENS Model 1080	12 (6)
		SIEMENS Biograph 128 mCT	42 (22)
		SIEMENS Biograph 40 mCT	74 (39)
		SIEMENS SOMATOM Definition AS mCT	2 (1)

		GE Discovery 710	7 (9)
		GE Discovery MI	3 (2)
		Philips GEMINI TF TOF 16	30 (16)
		Philips GEMINI TF TOF 64	1 (1)
		Philips Guardian Body(C)	8 (4)
**Imaging modality**	Which imaging modality was used in the study	FDG-PET scans post-treatment (up to 12 weeks after completion of neoadjuvant chemoradiotherapy)	10.3 weeks after neoadjuvant chemoradiotherapy [8.1, 11.2]
**Static/dynamic scans**	Static or dynamic scan	Static scan, 60 minutes after intravenous injection of ^{18}F-FDG	
	Acquisition time per time frame	2-3 minutes per bed position	
	Describe any temporal modelling technique that was used	-	
**Scanner calibration**	How and when the scanner was calibrated	Scanners were calibrated for EARL-1 measurements [7]	
**Patient instructions**	Specific instructions to patients during acquisition	Scanning in arms-up position	
**Anatomical motion correction**	Method used to minimise the effect of anatomical motion	-	
**Scan duration**	Duration of the complete scan	-	31 minutes [29, 36]
**Time-of-flight**	State if scanner time-of-flight capabilities are used during acquisition	Time-of-flight PET scanners were used	
**Reconstruction**			
**In-plane resolution**	Distance between pixels	-	4.1 mm [4.0, 4.1]
**Image slice thickness / image slice spacing**	Slice thickness	1.5 mm<br>mm<br>mm<br>mm	17 (9)<br>151 (80)<br>11 (6)<br>9 (5)
**Reconstruction method**	Reconstruction methods used in the different departments	BLOB-OS-TF	31 (16)
		LOR-RAMLA	8 (4)
		OSEM3D 3i24s	113 (60)
		OSEM3D 4i21s	12 (6)
		PSF+ TOF 2i21s	3 (2)
		PSF+ TOF 3i21s	2 (1)
		QCFX	2 (1)
		VPFX	4 (2)
		VPFXS	13 (7)
		VPHDS	1 (1)
		More detailed information on the number of iterations, subsets for iterative reconstruction, or other forms of correction, other than listed above, is not	

		available.
**Image registration**		
**Registration method**	Method used to register multi-modality imaging	Planning CT scans were rigidly registered to the ^{18}F-FDG PET scans using MIM Software version 7.1.3 (MIM Software Inc., Cleveland, OH, USA). The gross tumour volumes on planning CT scans were then be propagated onto the ^{18}F-FDG PET scans using the resultant registration vectors.
**Data conversion**		
**SUV normalization**	Which standardised uptake value (SUV) normalization method is used	SUV corrected for body weight and corrected for serum glucose
**Post-acquisition processing**		
		Detailed information on anti-aliasing, noise suppression, post-reconstruction smoothing filter, intensity normalization and other post- acquisition processing methods is not available.
**Segmentation**		
**Segmentation method**	How volumes of interest (VOI) were segmented	The gross tumour volume (GTV) available from the planning CT scans was used to determine the volume of interest (VOI). Planning CT scans were rigidly registered to the low dose CT scans belonging to the post-treatment ^{18}F-FDG PET scans. The VOIs on planning CT scans were transposed onto the low dose CT and ^{18}F-FDG PET scans using the resultant registration vectors. VOIs on the post-treatment scans were manually adapted to correct for tumour regression after nCRT (performed by M.J.V., in training, with 3 years of expertise in analysing ^{18}F-FDG PET scans in the current patient population). The resulting VOIs were revised in consensus by two investigators (M.J.V and R.J.B., who had 5 years of expertise)). Tumour delineations were done using the available pre- and post-treatment imaging, without using TRG outcome or any other clinical information.
**Conversion to mask**	Method to convert polygonal or mesh-based segmentations to a voxel-based mask	Crossing number algorithm
**Image interpolation**		
**Interpolation method**	Which interpolation algorithm was used to interpolate the image	Trilinear spline interpolation
	Position of the interpolation grid, *e.g.* align by centre	At the centre of the original grid
	Dimensions of the interpolation grid, *e.g.* rounded to nearest integer	Image intensities were not rounded
	Extrapolation beyond the original image	NaN values were returned
**Voxel dimensions**	Size of the interpolated voxels	2 x 2 x 2 mm. These voxel-dimensions were chosen to obtain a uniform isotropic voxel grid similarly to the development cohort. A uniform isotropic voxel grid is important since radiomic feature values are dependent on the tumour volume and the particular resolution, as well as the number of voxels involved in the calculation [8]. For the development cohort it was chosen to upsample original voxel dimensions (3.1819 x 3.1819 x 2 mm to 2 x 2 x 2 mm). Upsampling was preferred over downsampling: with upsampling information would be preserved at the cost of introducing artificial information. Downsampling would cause information loss and potential aliasing artifacts.

**ROI interpolation**		
**Interpolation method**	Which interpolation algorithm was used to interpolate the region of interest mask	Trilinear spline interpolation
**Partially masked voxels**	How partially masked voxels after interpolation are handled	Voxels with ≥50% coverage were included in the ROI
**Re-segmentation**		
**Re-segmentation methods**	Methods and settings to re-segment the ROI intensity mask	-
**Discretization**		
**Discretization method**	Method used to discretise image intensities	Fixed bin size: 0.5 g/mL, with lowest intensity at 0 g/mL
**Image transformation**		
**Image filter**	Methods and settings used to filter images, *e.g.* Laplacian-of-Gaussian	-
**Image biomarker computation**		
**Biomarker set**	Which set of image biomarkers is computed and refer to their definitions	The same set of features as reported previously [9].<br>In the development cohort, features were rescaled using min-max normalization. In order to apply the same method of rescaling in the external validation cohort, features of these patients were rescaled using the minimum and maximum value of the particular feature in the development cohort.
**IBSI compliance**	If the software used to extract the set of image biomarkers is compliant with the IBSI benchmarks	Yes, this was investigated previously [10].
**Robustness**	How robustness of the image biomarkers was assessed, *e.g.* test-retest analysis	Not performed in the external validation cohort. In the development cohort, features were tested for robustness by calculating an intraclass correlation coefficient (ICC) for slightly dilated delineations. The ICCs have been reported previously [9].
**Software**	Which software and version was used to compute image biomarkers	In-house developed software in Matlab 2014b (Mathworks, Natick, MA, USA)
**Image biomarker computation – texture parameters**		
**Texture matrix aggregation**	How texture-matrix based biomarkers were computed from underlying texture matrices	Matrices were merged over 3D directions before features were calculated
**Distance weighting**	How CM, RLM, NGTDM and NGLDM weight distances, *e.g.* no weighting	No weighting
**CM symmetry**	Whether symmetric or asymmetric co-occurrence matrices were computed	Symmetric
**CM distance**	The (Chebyshev) distance at	1

	which co-occurrence of intensities is determined, *e.g.* 1	
**SZM linkage distance**	Distance and distance norm for which voxels with the same intensity are considered to belong to the same zone for the purpose of construction an SZM, *e.g.* Chebyshev distance of 1	26-connectedness (linkage of a voxel to 26 neighbouring voxels with the same level) Chebyshev distance of 1
**NGTDM distance**	Neighbourhood distance and distance norm for the NGTDM, *e.g.* Chebyshev distance of 1	Chebyshev distance of 1

Supplemental Table 3. Radiomic quality score. Available from: www.radiomics.world/rqs

Image protocol quality - well-documented image protocols (for example, contrast, slice thickness, energy, etc.) and/or usage of public image protocols allow reproducibility/replicability

- [x] protocols well documented
- [x] public protocol used
- [ ] none

Multiple segmentations - possible actions are: segmentation by different physicians/algorithms/software, perturbing segmentations by (random) noise, segmentation at different breathing cycles. Analyse feature robustness to segmentation variabilities

- (•) yes
- ( ) no

Phantom study on all scanners - detect inter-scanner differences and vendor-dependent features. Analyse feature robustness to these sources of variability

- ( ) yes
- (•) no

Imaging at multiple time points - collect images of individuals at additional time points. Analyse feature robustness to temporal variabilities (for example, organ movement, organ expansion/shrinkage)

- ( ) yes
- (•) no

Feature reduction or adjustment for multiple testing - decreases the risk of overfitting. Overfitting is inevitable if the number of features exceeds the number of samples. Consider feature robustness when selecting features

- (•) Either measure is implemented
- ( ) Neither measure is implemented

Multivariable analysis with non-radiomics features (for example, EGFR mutation) - is expected to provide a more holistic model. Permits correlating/inferencing between radiomics and non radiomics features

- (•) yes
- ( ) no

Detect and discuss biological correlates - demonstration of phenotypic differences (possibly associated with underlying gene–protein expression patterns) deepens understanding of radiomics and biology

- ( ) yes
- (•) no

Cut-off analyses - determine risk groups by either the median, a previously published cut-off or report a continuous risk variable. Reduces the risk of reporting overly optimistic results

- (•) yes
- ( ) no

Discrimination statistics - report discrimination statistics (for example, C-statistic, ROC curve, AUC) and their statistical significance (for example, p-values, confidence intervals). One can also apply resampling method (for example, bootstrapping, cross-validation)

- [x] a discrimination statistic and its statistical significance are reported
- [x] a resampling method technique is also applied
- [ ] none

Calibration statistics - report calibration statistics (for example, Calibration-in-the-large/slope, calibration plots) and their statistical significance (for example, P- values, confidence intervals). One can also apply resampling method (for example, bootstrapping, cross-validation)

- [x] a calibration statistic and its statistical significance are reported
- [x] a resampling method technique is applied
- [ ] none

Prospective study registered in a trial database - provides the highest level of evidence supporting the clinical validity and usefulness of the radiomics biomarker

- ( ) yes
- (•) no

Validation - the validation is performed without retraining and without adaptation of the cut-off value, provides crucial information with regard to credible clinical performance

- [ ] No validation
- [ ] validation is based on a dataset from the same institute

- [ ] validation is based on a dataset from another institute
- [ ] validation is based on two datasets from two distinct institutes
- [x] the study validates a previously published signature
- [ ] validation is based on three or more datasets from distinct institutes

Comparison to 'gold standard' - assess the extent to which the model agrees with/is superior to the current 'gold standard' method (for example, TNM-staging for survival prediction). This comparison shows the added value of radiomics

- (•) yes
- ( ) no

Potential clinical utility - report on the current and potential application of the model in a clinical setting (for example, decision curve analysis).

- (•) yes
- ( ) no

Cost-effectiveness analysis - report on the cost-effectiveness of the clinical application (for example, QALYs generated)

- ( ) yes
- (•) no

Open science and data - make code and data publicly available. Open science facilitates knowledge transfer and reproducibility of the study

- [ ] scans are open source
- [ ] region of interest segmentations are open source
- [x] the code is open sourced
- [ ] radiomics features are calculated on a set of representative ROIs and the calculated features and representative ROIs are open source

Total score **21** (58.33%)

Supplemental Table 4. List of calculated radiomic features

Feature family	Image Biomarker Standardisation Initiative feature name
**morphologic features**	volume (mesh)
	volume (voxel counting)
	surface area (mesh)
	surface to volume ratio
	compactness 1
	compactness 2
	spherical disproportion
	sphericity
	asphericity
	centre of mass shift
	maximum 3D diameter
	major axis length
	minor axis length
	least axis length
	elongation
	flatness
	integrated intensity
	Morans I index
	Gearys C measure
**local intensity features**	local intensity peak
	global intensity peak
**intensity-based statistical features**	mean intensity
	intensity variance
	intensity skewness
	intensity kurtosis
	median intensity
	minimum intensity
	10th intensity percentile
	90th intensity percentile
	maximum intensity
	intensity interquartile range
	intensity range
	intensity-based mean absolute deviation
	intensity-based robust mean absolute deviation
	intensity-based median absolute deviation
	intensity-based coefficient of variation
	intensity-based quartile coefficient of dispersion
	intensity-based energy
	root mean square intensity

**grey level co-occurrence based features**	joint maximum
	joint average
	joint variance
	joint entropy
	difference average
	difference variance
	difference entropy
	sum average
	sum variance
	sum entropy
	angular second moment
	contrast
	dissimilarity
	inverse difference
	normalised inverse difference
	inverse difference moment
	normalised inverse difference moment
	inverse variance
	correlation
	autocorrelation
	cluster tendency
	cluster shade
	cluster prominence
	information correlation 1
	information correlation 2
**grey level run length based features**	short runs emphasis
	long runs emphasis
	low grey level run emphasis
	high grey level run emphasis
	short run low grey level emphasis
	short run high grey level emphasis
	long run low grey level emphasis
	long run high grey level emphasis
	grey level non uniformity
	normalised grey level non uniformity
	run length non-uniformity
	normalised run length non-uniformity
	run percentage
	grey level variance
	run length variance
	run entropy
**grey level size zone based features**	small zone emphasis
	large zone emphasis

	low grey level zone emphasis
	high grey level zone emphasis
	small zone low grey level emphasis
	small zone high grey level emphasis
	large zone low grey level emphasis
	large zone high grey level emphasis
	grey level non-uniformity
	normalised grey level non-uniformity
	zone size non-uniformity
	normalised zone size non-uniformity
	zone percentage
	grey level variance
	zone size variance
	zone size entropy
**neighbourhood grey tone difference based features**	coarseness
	contrast
	busyness
	complexity
	strength

Supplemental Table 5

Comparison of non-normalised radiomic feature values between the development cohort and external validation cohort

	**Development cohort (*n* = 73)** median [IQR]	**External validation cohort (*n* = 189)** median [IQR]	***P***
**joint maximum**	0.34 [0.25, 0.44]	0.17 [0.10, 0.23]	<.001
**median absolute deviation**	0.69 [0.52, 0.88]	0.52 [0.41, 0.68]	<.001
**joint entropy**	3.01 [2.46, 3.45]	4.22 [3.62, 4.85]	<.001
**sum entropy**	2.70 [2.24, 3.02]	3.30 [2.94, 3.67]	<.001
**angular second moment**	0.19 [0.14, 0.26]	0.08 [0.05, 0.11]	<.001
**inverse variance**	0.07 [0.05, 0.09]	0.45 [0.41, 0.48]	<.001

IQR = interquartile range

Supplemental Table 6

Distribution of clinical tumour (cT) stage versus tumour regression grade (TRG) for the development cohort and external validation cohort. Of the nine patients with cT1-2 stage in the development cohort, 7 (78%) had TRG 1. Of the 41 patients with cT1-2 stage in the external validation cohort, 12 had (29%) TRG 1 (*P* = .02).

**Development cohort**		**TRG 1**	**TRG 2-3-4**
	**cT1-2**	7	2
	**cT3-4a**	9	55
**External validation cohort**		**TRG 1**	**TRG 2-3-4**
	**cT1-2**	12	29
	**cT3-4a**	28	113

Supplemental Table 7

Coefficients of LASSO models based on the combined development and external validation cohorts.

Outcome	LASSO model	Feature name	Coefficient
**TRG 2-3-4**	radiomic features + clinical variables	intercept	2.11
		cT stage<br>cT3-4a<br>cT1-2	 0.22<br>1 (ref)
		histology<br>squamous cell carcinoma adenocarcinoma	 -0.96<br>1 (ref)
		Gearys C measure	0.019
		least axis length	0.057
		quartile coefficient of dispersion	0.16
	clinical variables	intercept	3.16
		cT stage<br>cT3-4a<br>cT1-2	 0.65<br>1 (ref)
		age	-0.019
		histology<br>squamous cell carcinoma adenocarcinoma	 -1.40<br>1 (ref)
**TRG 3-4**	radiomic features + clinical variables	intercept	-0.375
		cT stage<br>cT3-4a<br>cT1-2	 0.262<br>1 (ref)
		histology<br>squamous cell carcinoma adenocarcinoma	 -0.083<br>1 (ref)
		intensity-based coefficient of variation	0.070
		flatness	0.128
		Gearys C measure	0.067
		least axis length	0.073
		minimum	-0.019
		run entropy	0.023
		surface to volume ratio	-0.094
	clinical variables	intercept	-0.805
		cT stage<br>cT3-4a<br>cT1-2	 0.590<br>1 (ref)
		sex<br>male<br>female	 0.164<br>1 (ref)
		histology<br>squamous cell carcinoma adenocarcinoma	 -0.472<br>1 (ref)

LASSO = least absolute shrinkage and selection operator; cT = clinical tumour stage

Supplemental Table 8

Names of radiomic features with AUCs of at least 0.60 that were selected after the univariable feature selection step was applied to the combined cohorts for model extension (see Appendix 1 for details). The median area under the receiver operating characteristic curve (AUC) is shown for the 100 training datasets.

Outcome	Feature name	Median AUC over 100 training splits
**TRG 2-3-4**	surface to volume ratio	0.67
	run length non-uniformity	0.67
	coefficient of variation	0.65
	information correlation 2	0.65
	angular second moment	0.65
	flatness	0.62
	local intensity peak	0.61
	Gearys C measure	0.61
**TRG 3-4**	surface to volume ratio	0.65
	flatness	0.62
	run length non-uniformity	0.62
	intensity variance	0.60
	intensity skewness	0.60

Supplemental Table 9

Four machine learning models were explored after univariable feature selection (see Appendix 1 for details). Three linear machine learning models and one non-linear model were evaluated over 100 training and validation datasets (2:1 ratio) using selected radiomic features (Supplemental Table 7) plus the clinical variables clinical tumour and node stage, sex, age and histology. Performance metrics for the 100 training and validation datasets are shown in the table.

Outcome	Model type		AUC mean (95% CI)	Sensitivity (%) mean (95% CI)	Specificity (%) mean (95% CI)	Accuracy (%) mean (95% CI)
**TRG 2-3-4**	**logistic regression**	training	0.76 (0.68 – 0.81)	68 (47 – 88)	74 (49 – 95)	69 (57 – 81)
		validation	0.70 (0.58 – 0.83)	69 (40 – 95)	71 (33 – 94)	69 (51 – 85)
	**SVM**	training	0.72 (0.67 – 0.77)	77 (68 – 87)	67 (51 – 78)	75 (69 – 81)
		validation	0.65 (0.57 – 0.74)	74 (59 – 85)	57 (36 – 78)	70 (62 – 79)
	**Naïve Bayes**	training	0.77 (0.73 – 0.82)	73 (54 – 86)	75 (57 – 92)	73 (62 – 80)
		validation	0.73 (0.63 – 0.84)	71 (49 – 92)	74 (50 – 94)	72 (58 – 84)
	**random forest***	training	0.81	83	78	82
**TRG 3-4**	**logistic regression**	training	0.71 (0.65 – 0.76)	73 (51 – 88)	63 (45 – 81)	68 (63 – 72)
		validation	0.63 (0.50 – 0.73)	73 (40 – 95)	54 (29 – 85)	64 (57 – 72)
	**SVM**	training	0.65 (0.61 – 0.70)	74 (60 – 84)	57 (42 – 71)	65 (61 – 70)
		validation	0.58 (0.51 – 0.68)	66 (44 – 82)	50 (29 – 69)	58 (51 – 68)
	**Naïve Bayes**	training	0.69 (0.65 – 0.73)	77 (62 – 90)	57 (41 – 72)	67 (63 – 71)
		validation	0.65 (0.54 – 0.74)	76 (48 – 95)	55 (26 – 85)	66 (58 – 73)
	**random forest***	training	0.75	77	73	75

* The random forest was fitted on one dataset consisting of both cohorts, which means that no performance distributions are available.

AUC = area under the receiver operating characteristic curve; IQR = interquartile range; SVM = support vector machine; 95% CI = 95% confidence interval

Supplemental Figures

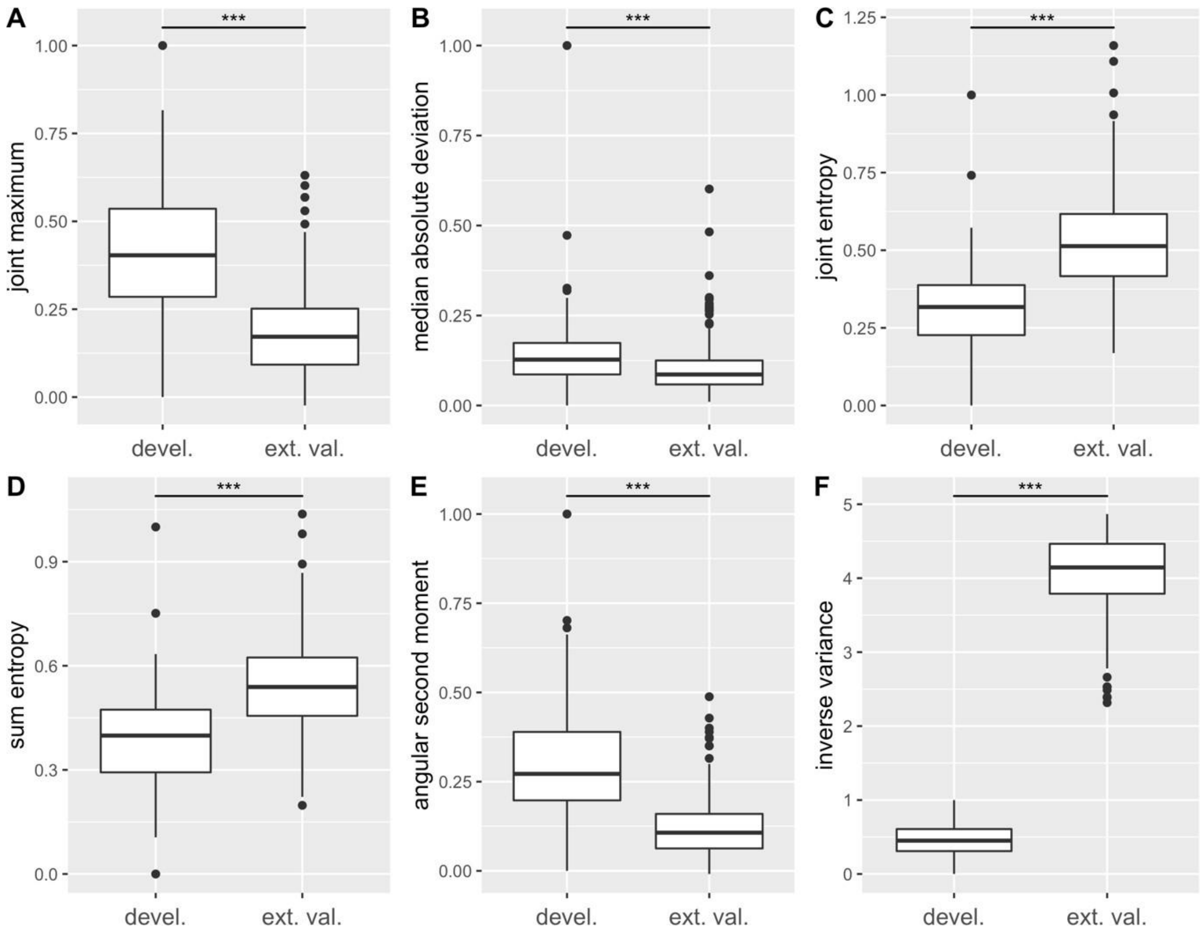

Supplemental Figure 1. Comparison of radiomic feature values, after rescaling (y-axis), between the development cohort and external validation cohort (x-axis). For the external validation cohort rescaling was done with the minimum and maximum values of the development cohort.

devel. = development cohort; ext. val. = external validation cohort

*** $P < .001$

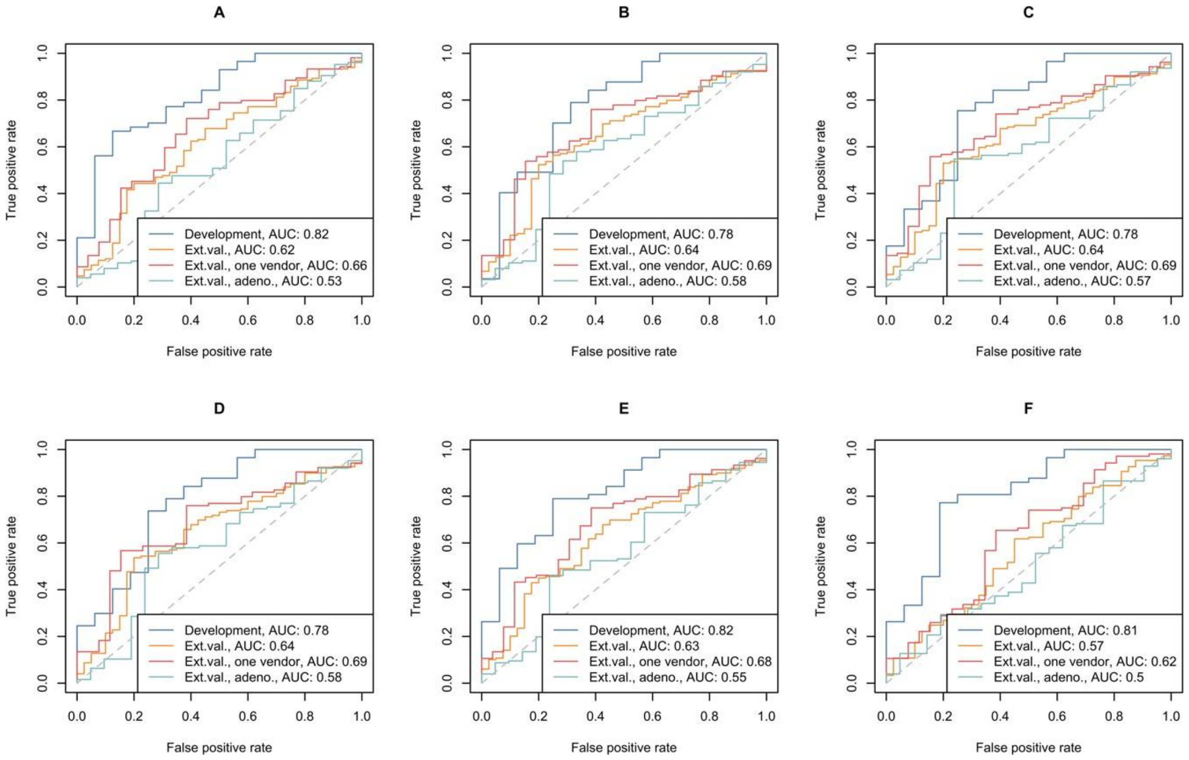

Supplemental Figure 2. ROC curves for the six externally validated models (A-F). The blue line corresponds to the development cohort (*n* = 73), the orange line to the external validation cohort (*n* = 189), the red line to the external validation cohort when scans from one vendor (SIEMENS, *n* = 130) were included, and the green line to the external validation cohort when the external validation cohort was limited to only adenocarcinoma patients (*n* = 147).

ROC = Receiver Operating Characteristic curve; Development = development cohort; Ext. val. = external validation cohort

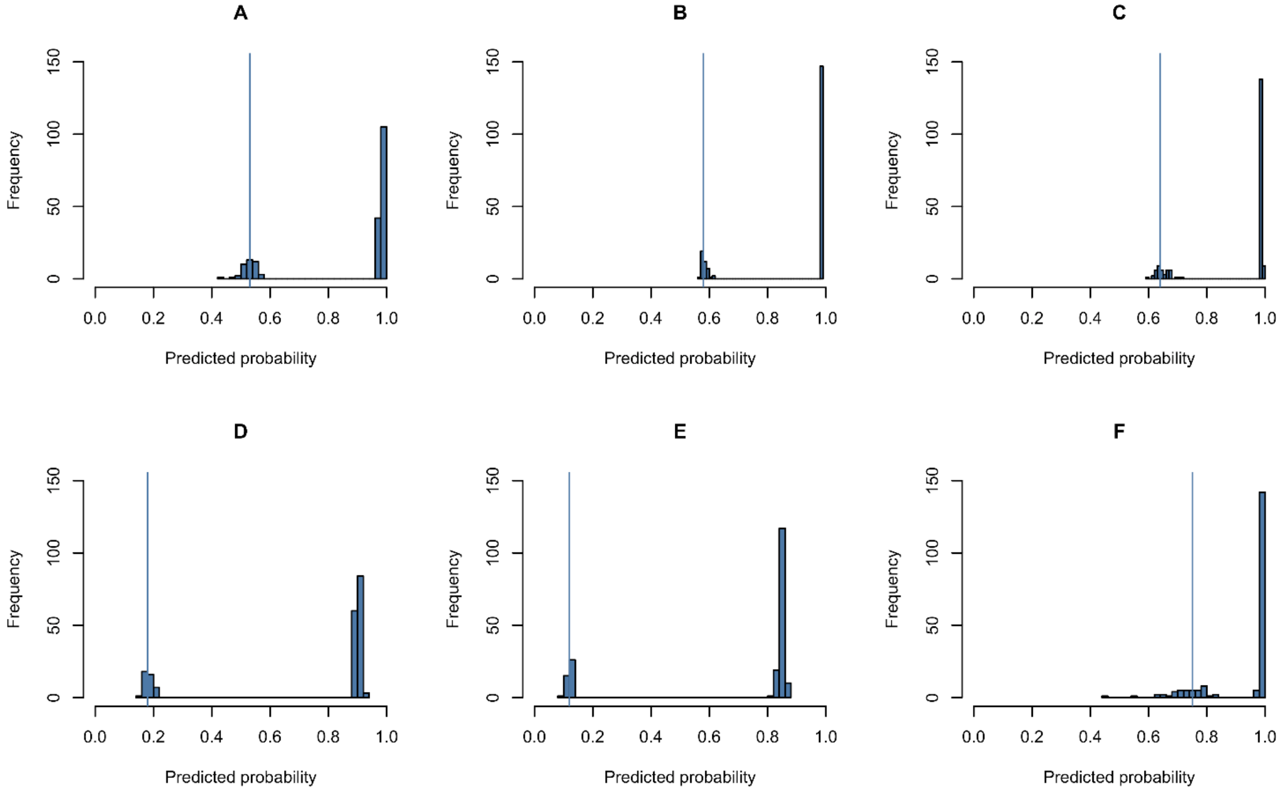

Supplemental Figure 3. Histograms of predicted probabilities for TRG 2-3-4 with models A-F as applied onto the external validation cohort. The blue line shows the probability threshold as determined to obtain the benchmark of 90% sensitivity [11]. Predicted probabilities near 1.0 correspond to prediction of TRG 2-3-4, predicted probabilities near 0.0 correspond to prediction of TRG 1. In all six models, a threshold chosen between the two groups of observations in the histogram (*e.g.* at 0.8 for model A) completely separates patients based on clinical tumour stage (cT) (*i.e.* in model A all patients with cT1-2 have predicted probabilities <0.8 and all patients with cT3-4a have predicted probabilities >0.8).

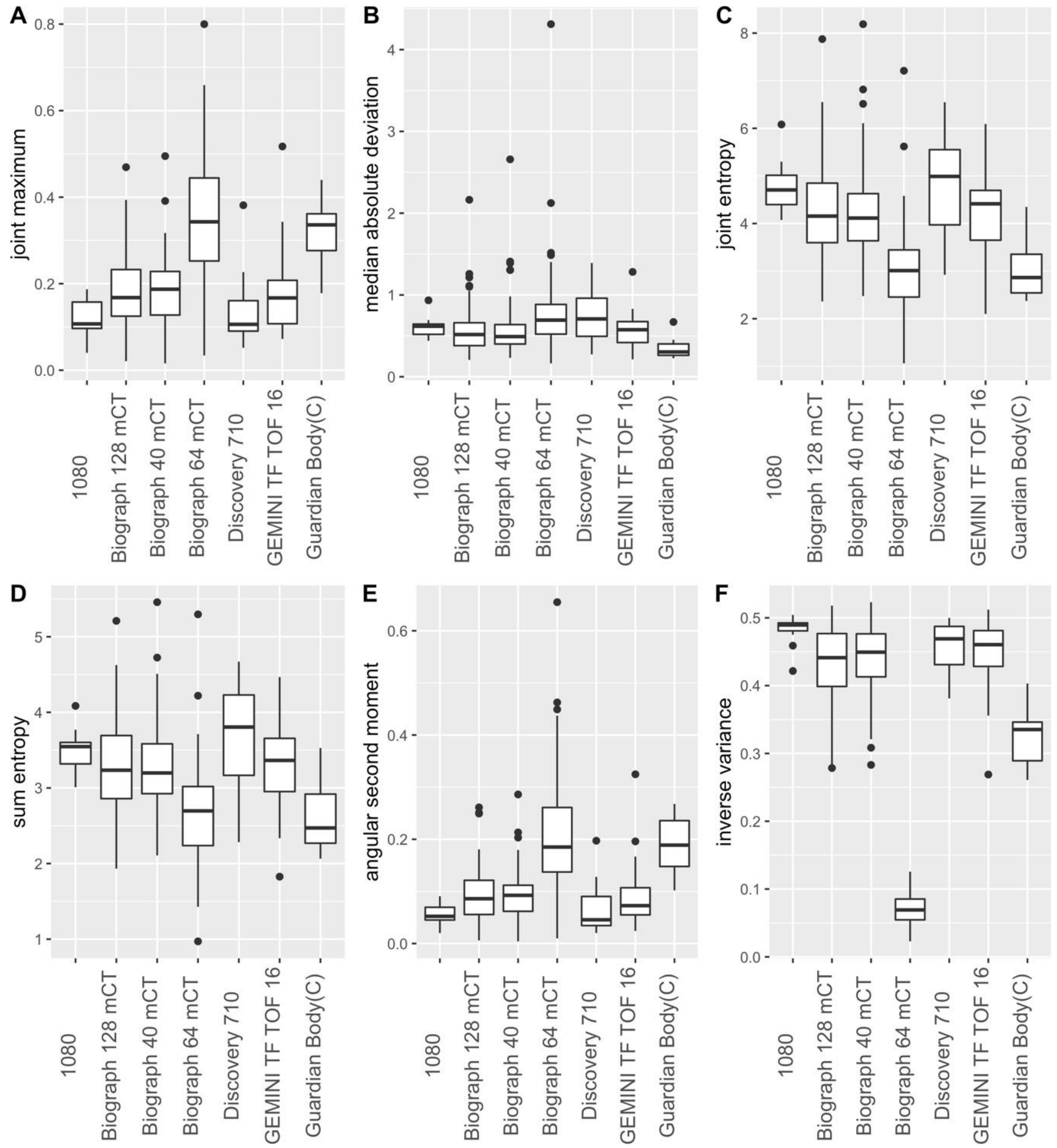

Supplemental Figure 4. Boxplots demonstrating dependency of radiomic feature values (y- axis) on scanner types (x-axis) for the six radiomic features that were used in the externally validated prediction models (A-F). Boxplots show the median and interquartile range for non- normalised radiomic features which were calculated in the combined cohorts (*i.e.* development cohort and external validation cohort combined).

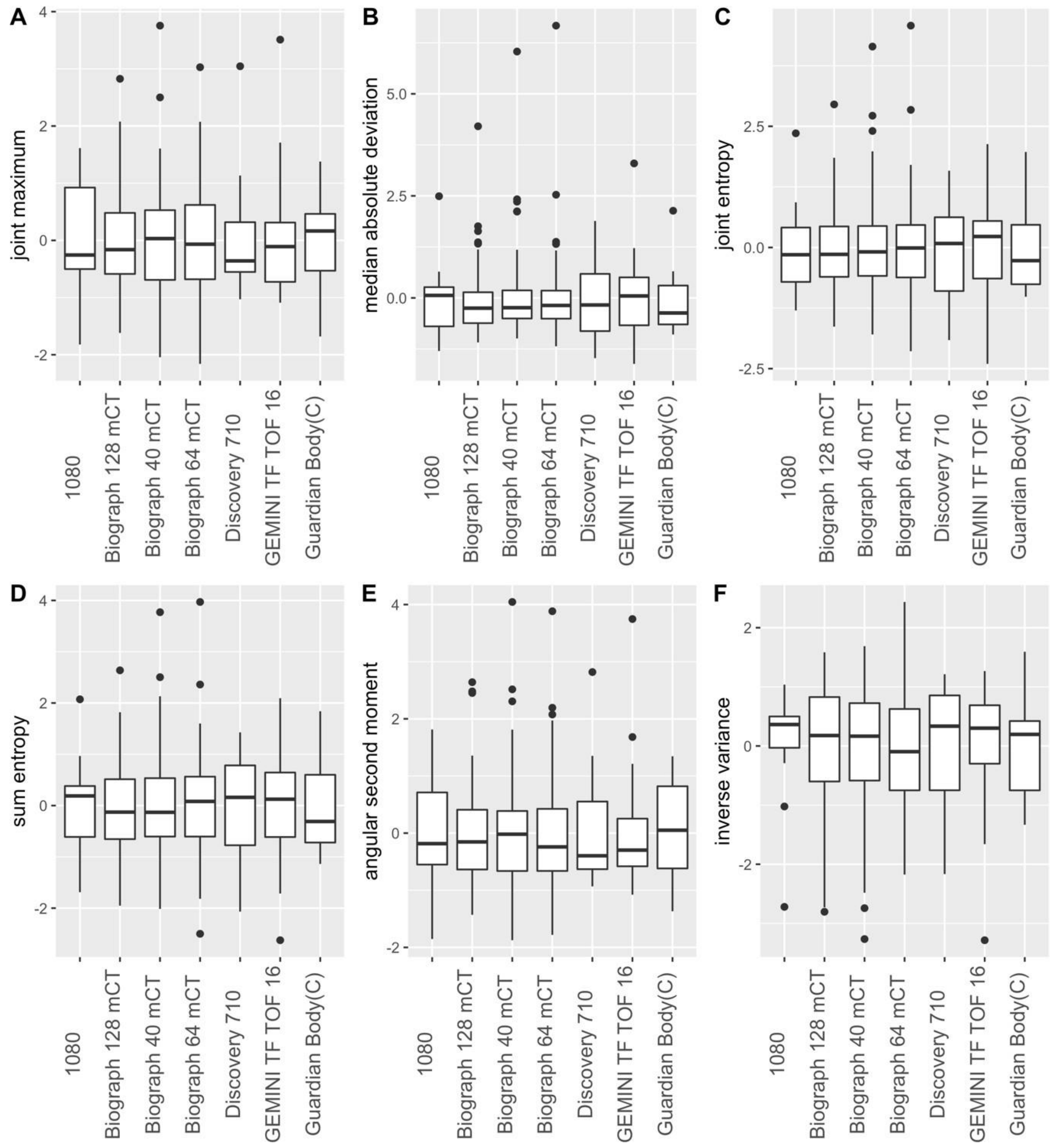

Supplemental Figure 5. Boxplots show the median and interquartile range for the six radiomic features (y-axis) that were used in the externally validated prediction models (A-F) after scanner-specific standardisation was performed. Scanner-specific standardisation was applied on the combined cohorts (*i.e.* development cohort and external validation cohort combined). The names of the different scanner types are shown on the x-axis.

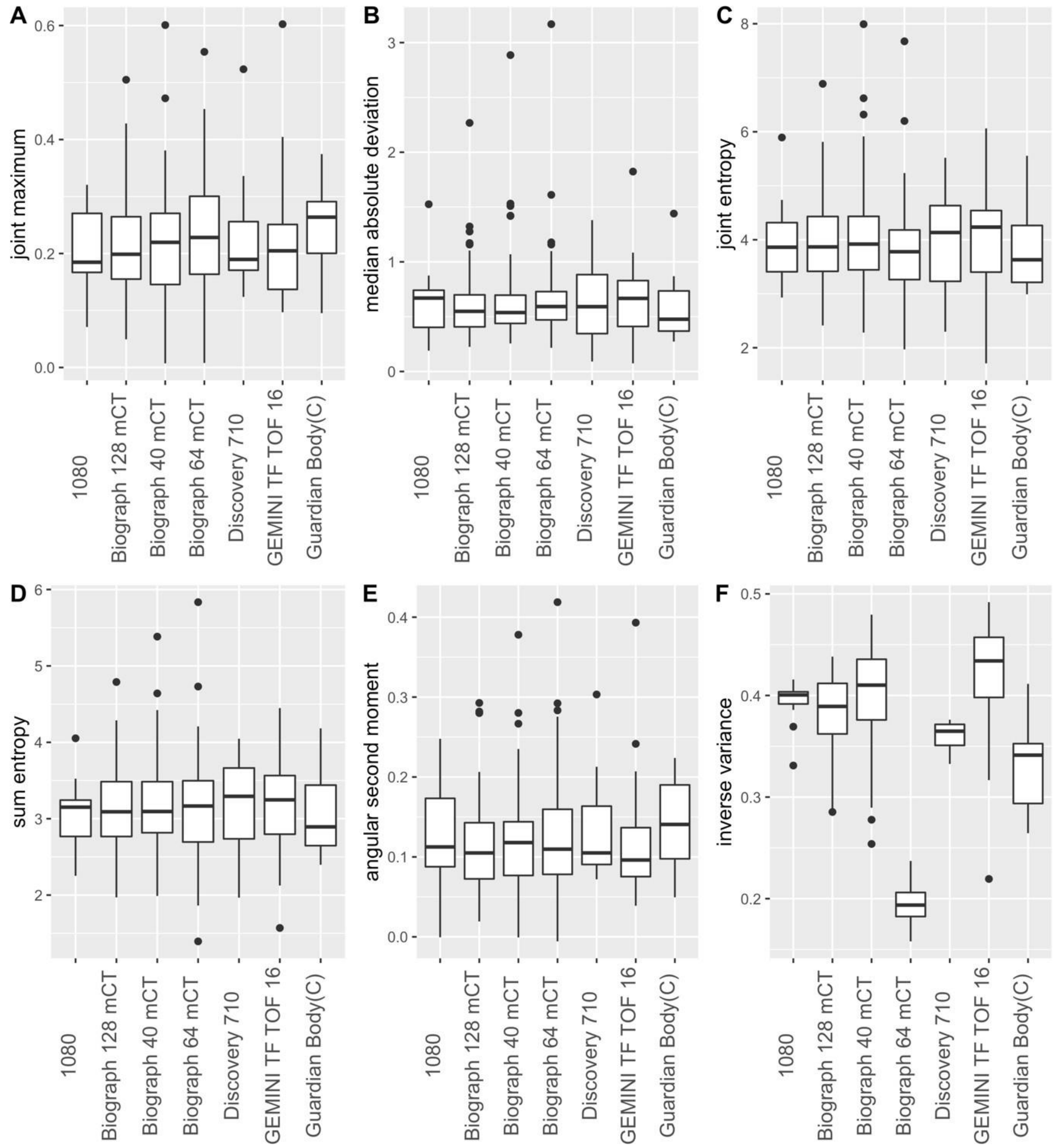

Supplemental Figure 6. Boxplots show the median and interquartile range for the six radiomic features (y-axis) that were used in the externally validated prediction models (A-F) after ComBat harmonization was performed. ComBat harmonization was applied on the combined cohorts (*i.e.* development cohort and external validation cohort combined). The names of the different scanner types are shown on the x-axis.

A

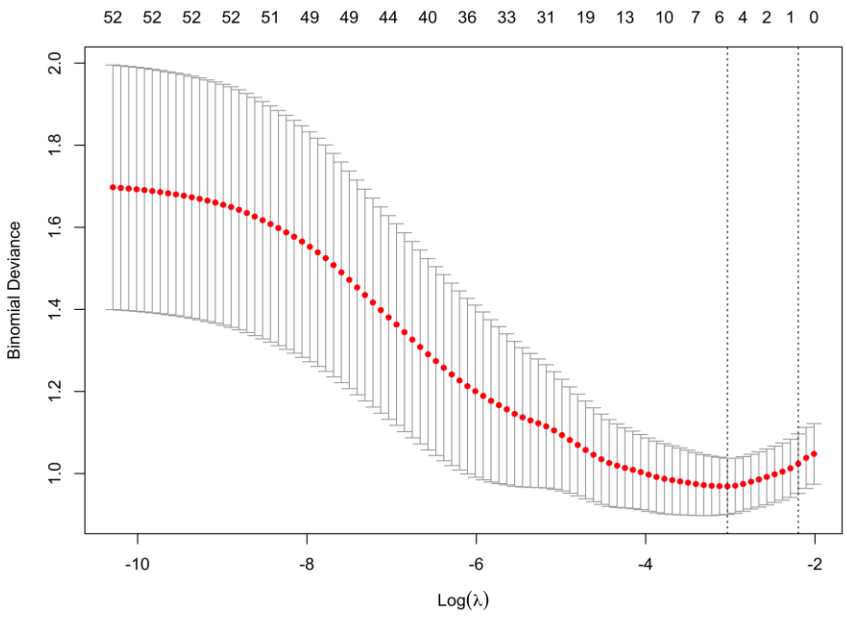

B

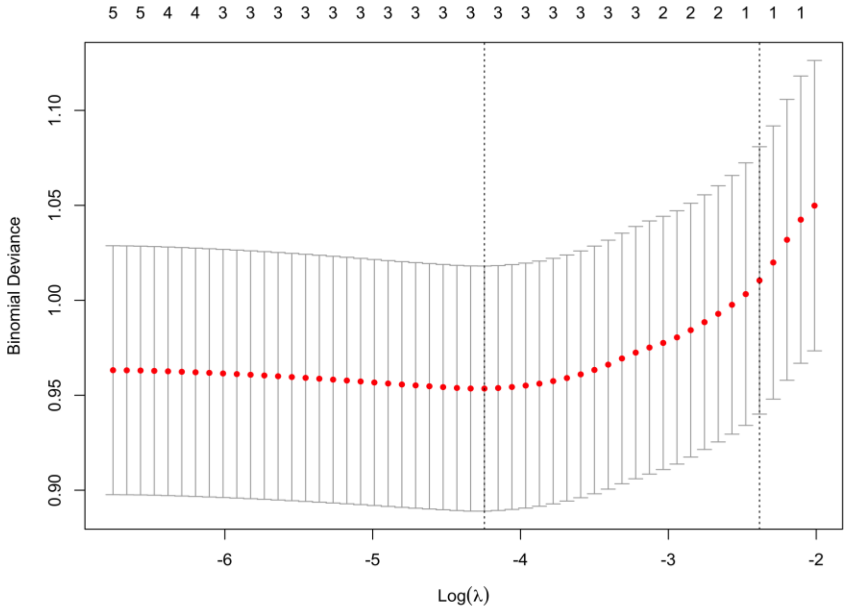

Supplemental Figure 7. Values of log(λ) (x-axis) versus the binomial deviance (y-axis) for (A) the extended LASSO model to detect TRG 2-3-4 including radiomic features and clinical variables and (B) the LASSO model including clinical variables only. The log(λ) (vertical line) was chosen at a value to minimise binomial deviance. The number of variables with a non- zero coefficient that correspond to the log(λ) on the x-axis is shown at the top of the plot.

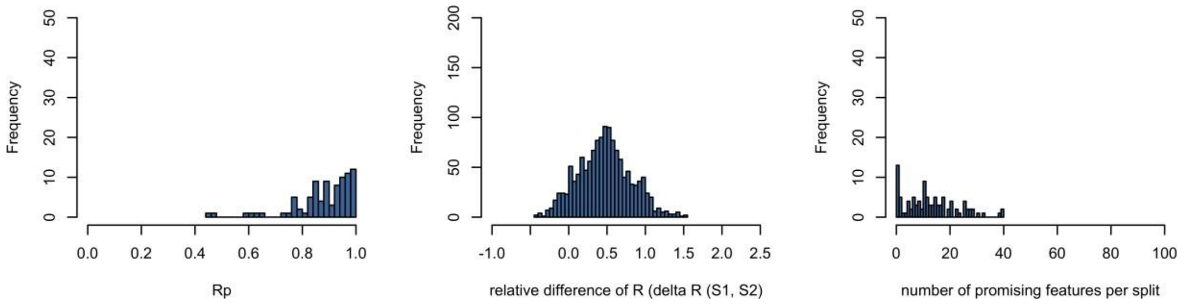

Supplemental Figure 8. Histogram of 100 R_p values (obtained from 100 splits) for post-nCRT ^{18}F-FDG PET features in the combined datasets (*i.e.* development cohort and external validation cohort combined). Mean R_p values below 0.0 imply generic radiomic analysis is unsuitable.

REFERENCES

1. Steyerberg EW. Clinical prediction models: a practical approach to development, validation, and updating. New York: Springer, 2009.
2. Chatterjee A, Vallières M, Dohan A, et al. Creating robust predictive radiomic models for data from independent institutions using normalization. *IEEE Trans Radiat Plasma Med Sci* 2019;3:210-5.
3. Orlhac F, Boughdad S, Philippe C, et al. A postreconstruction harmonization method for multicenter radiomic studies in PET. *J Nucl Med* 2018;59:1321-8.
4. Johnson WE, Li C, Rabinovic A. Adjusting batch effects in microarray expression data using empirical Bayes methods. *Biostatistics (Oxford, England)* 2007;8:118-27.
5. Chatterjee A, Vallières M, Dohan A, et al. An empirical approach for avoiding false discoveries when applying high-dimensional radiomics to small datasets. *IEEE Trans Radiat Plasma Med Sci* 2019;3:201-9.
6. Toxopeus EL, Nieboer D, Shapiro J, et al. Nomogram for predicting pathologically complete response after neoadjuvant chemoradiotherapy for oesophageal cancer. *Radiother Oncol* 2015;115:392-8.
7. Boellaard R, O'Doherty MJ, Weber WA, et al. FDG PET and PET/CT: EANM procedure guidelines for tumour PET imaging: version 1.0. *Eur J Nucl Med Mol Imaging* 2010;37:181-200.
8. Yan J, Chu-Shern JL, Loi HY, et al. Impact of image reconstruction settings on texture features in 18F-FDG PET. *J Nucl Med* 2015;56:1667-73.
9. Beukinga RJ, Hulshoff JB, Mul VEM, et al. Prediction of response to neoadjuvant chemotherapy and radiation therapy with baseline and restaging (18)F-FDG PET imaging biomarkers in patients with esophageal cancer. *Radiology* 2018;287:983-92.
10. Zwanenburg A, Vallières M, Abdalah MA, et al. The Image Biomarker Standardization Initiative: standardized quantitative radiomics for high-throughput image-based phenotyping. *Radiology* 2020;295:328-38.
11. Noordman BJ, Spaander MCW, Valkema R, et al. Detection of residual disease after neoadjuvant chemoradiotherapy for oesophageal cancer (preSANO): a prospective multicentre, diagnostic cohort study. *Lancet Oncol* 2018;19:965-74.
